# Finite-Element Model Predicts Current Density Distribution for Clinical Applications of tDCS and tACS

**DOI:** 10.3389/fpsyt.2012.00083

**Published:** 2012-09-24

**Authors:** Toralf Neuling, Sven Wagner, Carsten H. Wolters, Tino Zaehle, Christoph S. Herrmann

**Affiliations:** ^1^Experimental Psychology Lab, University of OldenburgOldenburg, Germany; ^2^Institute for Biomagnetism and Biosignalanalysis, University of MünsterMünster, Germany; ^3^Department of Neurology, Section of Neuropsychology, Otto-von-Guericke UniversityMagdeburg, Germany; ^4^Research Center Neurosensory Science, University of OldenburgOldenburg, Germany

**Keywords:** tDCS, tACS, finite-element modeling

## Abstract

Transcranial direct current stimulation (tDCS) has been applied in numerous scientific studies over the past decade. However, the possibility to apply tDCS in therapy of neuropsychiatric disorders is still debated. While transcranial magnetic stimulation (TMS) has been approved for treatment of major depression in the United States by the Food and Drug Administration (FDA), tDCS is not as widely accepted. One of the criticisms against tDCS is the lack of spatial specificity. Focality is limited by the electrode size (35 cm^2^ are commonly used) and the bipolar arrangement. However, a current flow through the head directly from anode to cathode is an outdated view. Finite-element (FE) models have recently been used to predict the exact current flow during tDCS. These simulations have demonstrated that the current flow depends on tissue shape and conductivity. To face the challenge to predict the location, magnitude, and direction of the current flow induced by tDCS and transcranial alternating current stimulation (tACS), we used a refined realistic FE modeling approach. With respect to the literature on clinical tDCS and tACS, we analyzed two common setups for the location of the stimulation electrodes which target the frontal lobe and the occipital lobe, respectively. We compared lateral and medial electrode configuration with regard to their usability. We were able to demonstrate that the lateral configurations yielded more focused stimulation areas as well as higher current intensities in the target areas. The high resolution of our simulation allows one to combine the modeled current flow with the knowledge of neuronal orientation to predict the consequences of tDCS and tACS. Our results not only offer a basis for a deeper understanding of the stimulation sites currently in use for clinical applications but also offer a better interpretation of observed effects.

## Introduction

In the past years, non-invasive brain stimulation techniques have gained interest in the treatment of psychiatric and neurological disorders. Especially repetitive TMS (rTMS) and transcranial electrical stimulation (TES), which comprises tDCS as well as tACS, have proven to be successful candidates as tools for therapeutic treatment. However, while TMS has been approved for treatment of major depression by the FDA, the promising results of tDCS-studies on the treatment of neurological and psychiatric diseases have not be put into everyday practice (George and Aston-Jones, 2010). Numerous studies have shown that tDCS is feasible for a wide range of disorders, e.g., motor disorders after stroke (Hummel et al., 2005; Edwards et al., 2009), post stroke aphasia (Monti et al., 2008; Baker et al., 2010), epilepsy (Fregni et al., 2006b; Nitsche and Paulus, 2009), chronic pain (Boggio et al., 2009), Parkinson’s disease (Boggio et al., 2006; Benninger et al., 2010), and Alzheimer’s disease (Ferrucci et al., 2008; Boggio et al., 2011). Furthermore, tDCS has demonstrated its potential to modulate working memory performance which could be used to treat neuropsychiatric deficits (Zaehle et al., 2011; Heimrath et al., 2012). Appealing characteristics of TES comprise cost, usability, and sham-control. TES devices are affordable, compared to TMS, weigh less than 1 kg and can easily be used at home or as a mobile device. Additionally, tDCS can easily be sham-controlled and has mostly well-tolerated, mild adverse effects.

Because some neurological and psychiatric disorders involve altered brain activity, the potential of tDCS to modulate this activity is obvious. Electrical stimulation is a candidate to facilitate impaired function or to suppress maladaptive plasticity (Paulus, 2011). TDCS uses a direct current with low intensity which is passed into the brain via scalp electrodes. Spontaneous neural activity is modulated in a polarity dependent manner (Bindman et al., 1964; Nitsche and Paulus, 2009). Anodal tDCS leads to a depolarization of the resting membrane potential and enhances cortical excitability whereas cathodal tDCS leads to a hyperpolarization and a reduction of cortical excitability. These effects can outlast the stimulation for an hour or even longer, given sufficient duration of stimulation (Nitsche and Paulus, 2000, 2001). Other electrical stimulation techniques use oscillating currents (Marshall et al., 2006; Antal et al., 2008; Kanai et al., 2008; Zaehle et al., 2010). TACS and tACS with an offset called oscillating transcranial direct current stimulation (otDCS) are able to modulate the ongoing rhythmic brain activity and could be used to treat diseases that are associated with disturbed brain oscillations (Herrmann and Demiralp, 2005; Uhlhaas et al., 2008). It can be concluded, that tDCS and tACS offer a wide range of possible therapeutic application and their potential has already been demonstrated in numerous pilot studies. Two of the major target areas of these studies were the visual cortex and the frontal cortex.

Targeting the visual cortex with tDCS and tACS could be beneficial for diseases with deficient visual processing and changes in excitability of visual areas (e.g., amblyopia, migraine, and neglect; Antal et al., 2004). Halko et al. (2011) demonstrated with a case study that tDCS facilitated rehabilitation of hemianopia. Additionally, Sabel et al. (2011) have successfully applied peripheral ACS to the visual system in the restoration of visual function in patients with optic neuropathy. They also reported long lasting EEG changes in the occipital cortex after stimulation. Furthermore, studies on healthy volunteers with tDCS (Antal et al., 2003; Antal and Paulus, 2008; Chaieb et al., 2008) and tACS (Kanai et al., 2008; Zaehle et al., 2010) have proven their capability to modulate excitability of the visual cortex.

The frontal cortex has been stimulated to manipulate excitability in different areas. For example, anodal stimulation aiming at the left frontal gyrus, which is essential for speech production (Hillis et al., 2004), facilitated this function (Baker et al., 2010; Fridriksson et al., 2011; Holland et al., 2011; Marangolo et al., 2011). The authors argued that this design could be used to treat aphasic stroke patients. In major depression, pathologically altered activity of the prefrontal cortex has been demonstrated. Compared to the right dorsolateral prefrontal cortex (dlPFC), the left dlPFC is hypoactivated (Grimm et al., 2008). A bilateral frontal application of the tDCS electrodes would therefore be very convenient to achieve a balance of left and right dlPFC, i.e., excitability enhancement of the left dlPFC and an excitability reduction of the right dlPFC. Studies on the antidepressive effect of prefrontal tDCS provided promising results (see Nitsche and Paulus, 2009 for a review; Kalu et al., 2012 for a meta-analysis). Major advantage of tDCS in depression therapy might be its immediate effect compared to the delayed effect of depression pharmacotherapy (Rigonatti et al., 2008) and its benefits for patients who do not respond to pharmaceutical interventions (Dell’Osso et al., 2011).

An important aspect of the mechanisms of TES is the magnitude and location of the induced current. Although tDCS is rather non-focal, the locations of the stimulation electrodes are critical for the amount of current being shunted through the scalp, how much is delivered to the brain, and to what regions (Miranda et al., 2006; Feira et al., 2009). In clinical contexts, it is crucial to know if the electrode positions are suited to induce an electric field in the target brain area and furthermore, if the induced current is of sufficient magnitude. A further critical aspect of tDCS is the direction of the current flow with regard to the neuronal orientation in space (Nitsche and Paulus, 2000). To address these issues, we used a realistic finite-element modeling approach on lateral and medial electrode configurations targeting the occipital or frontal cortex. Our results allow for high resolution insights into the current flow in the targeted brain areas and are discussed with respect to their usability.

## Materials and Methods

### tDCS simulation

**Figure 1:**
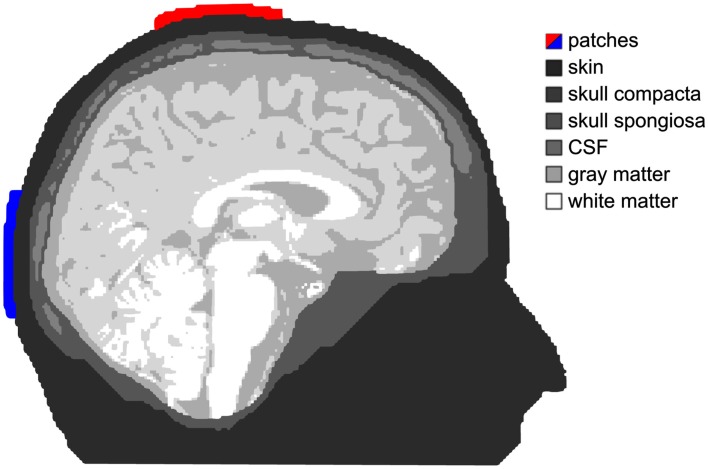
**Tissue compartments**. Shades of gray depict the different tissue compartments used in our simulation. The anode is colored red and the cathode is blue. This applies for all simulation figures.

For a tDCS simulation study, a realistic FE model of the head was generated from a T1-weighted, a T2-weighted and a diffusion-tensor (DT)-magnetic resonance image (MRI) of a healthy 26-year-old male subject. In a first step, the T2-MRI was rigidly registered onto the T1-MRI using a mutual information based cost-function (Jenkinson and Smith, 2001). Segmentation into tissue compartments skin, skull compacta, skull spongiosa, cerebrospinal fluid (CSF), brain gray (GM), and white matter (WM) was then performed using the FSL software^1^ (Zhang et al., 2001; Smith, 2002; Jenkinson et al., 2005). Segmentation started with the generation of initial masks for skin, inner and outer skull, and brain using both T1- and T2-MRI. In a second step, the T1-MRI served for GM and WM segmentation, while the T2-MRI allowed for a segmentation of skull compacta, skull spongiosa, and CSF space (see Figure 1). For the compartments of skin, skull compacta, skull spongiosa, and CSF, we used the isotropic conductivity values of 0.43, 0.007, 0.025, and 1.79 S/m, respectively (Baumann et al., 1997; Akhtari et al., 2002; Dannhauer et al., 2011). For the modeling of white matter conductivity anisotropy, the diffusion-weighted images were first artifact-corrected using our reversed-gradient approach introduced in Olesch et al. (2010). Diffusion tensors were then determined and the result was registered onto the structural images using the FSL routine vecreg^2^. In a last step, the conductivity anisotropy in GM and WM was computed from the registered DTI using an effective medium approach (Tuch et al., 2001; Rullmann et al., 2009). This resulted in mean conductivities of 0.19 and 0.24 S/m for WM and GM.

Two electrode patches with a size of 7 cm × 5 cm, thickness of 4 mm, and saline like conductivity of 1.4 S/m are modeled. A total current of 1 mA is injected at the red patch (anode) and removed at the blue one (cathode). For field modeling of this stimulation throughout the volume conductor, a quasistatic approximation of Maxwell’s equations (Plonsey and Heppner, 1967) was used, resulting in a Laplace equation for the electric potential Φ with inhomogeneous Neumann boundary conditions at the head surface. An isoparametric FE approach is used for the computation of Φ in a 1 mm geometry-adapted hexahedral mesh (Wolters et al., 2007a,b), resulting in a large sparse linear equation system with 2.255 million unknowns, which is solved using an algebraic multigrid preconditioned conjugate gradient approach (Wolters et al., 2002; Lew et al., 2009). In a last step, the current density *J* = *σ* grad Φ is computed with *σ* being the 3 × 3 conductivity tensor. For simulation, we used our software SimBio^3^.

In all subsequent figures, slices of the current density distribution through the cortex are presented. The amplitude of *J* is coded by means of a linear color-scale. The current density distributions were computed in the 1 mm geometry-adapted hexahedral head model and the software SciRun^4^ was used for visualization.

## Results

### General findings

The strongest current flow was observed within the skin (peak current density 1.7 A/m^2^), since the conductivity of skin is much better than that of bone. For this reason, current densities in skin were not visualized. Otherwise, the pattern of the intracranial currents could not be made visible. Inside the skull, conductivity of cerebrospinal fluid is again much better than that of GM and WM. Thus, also current densities within CSF (peak current density 0.378 A/m^2^) are not visualized.

A general phenomenon that could be observed for the current densities in GM and WM was the fact that cerebral regions adjacent to CSF showed stronger current densities than more remote areas. Gyri and small structures that protrude into CSF receive strongest current densities. Current densities within gray and white matter were in the range of 0–0.1 A/m^2^.

### Electrode montage FPz/Oz

**Figure 2:**
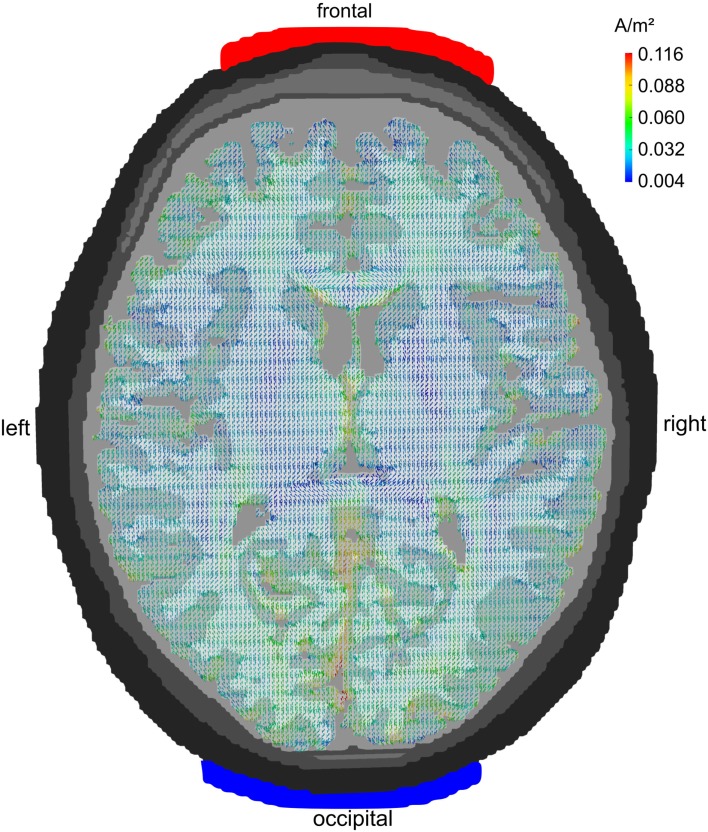
**Midline configuration FPz/Oz: axial view**. Stimulation electrodes are centered around electrode positions FPz and Oz. The current density is not very focal but rather widespread. All cortical lobes show current densities above 0.07 A/m^2^. The maximum current flow is in the occipital cortex.

Figure 2 depicts the current densities when stimulation electrodes are centered around 10–20-electrode positions FPz and Oz. As can be seen from the figure, current flow is rather widespread and reaches all cortical lobes. The montage is not ideal for a selective stimulation of the frontal lobe. Occipital cortex receives stronger currents than frontal cortex – albeit mainly in gyri adjacent to the interhemispheric cleft as current density is generally strong in those areas of GM that lie close to CSF.

### Electrode montage F7/F8

**Figure 3:**
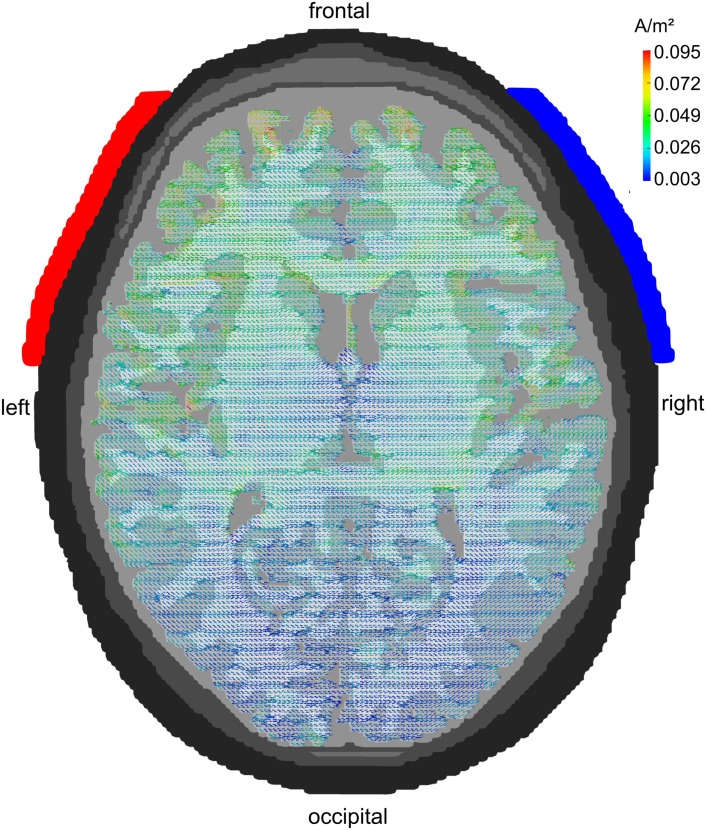
**Lateral configuration F7/F8: axial view**. Stimulation electrodes are centered around electrode positions F7 and F8. The current density is clearly localized to the frontal cortex. Temporal and parietal lobes show only weak current densities and the occipital lobe receives hardly any stimulation.

Figure 3 displays the pattern of current densities for the electrodes being centered around 10–20-electrode positions F7 and F8. Current flow is not restricted to but focused to frontal regions. Temporal and parietal cortex receive significantly weaker stimulation and occipital cortex almost none. This montage is, therefore, well suited to stimulate frontal brain areas without too much involvement of other cortical lobes.

### Electrode montage Cz/Oz

**Figure 4:**
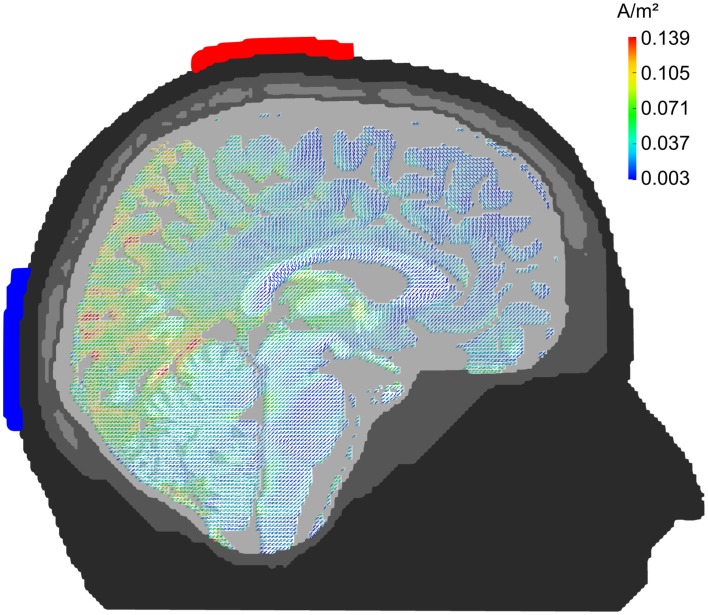
**Midline configuration Cz/Oz: sagittal view**. Stimulation electrodes are centered around electrode positions Cz and Oz. The pattern of current densities shows a clear maximum in posterior brain areas, especially in occipital cortex.

Figure 4 depicts the pattern of current densities when stimulation electrodes are centered around 10–20-electrode positions Cz and Oz. Parietal and occipital cortex show strong current densities in the range of 0.05–0.15 A/m^2^. Frontal brain regions receive significantly less stimulation current with the exception of the orbito-frontal cortex. The montage seems well suited for occipital stimulation. Note, however, that current density is stronger in medial than in lateral occipital cortex.

### Electrode montage P7/P8

**Figure 5:**
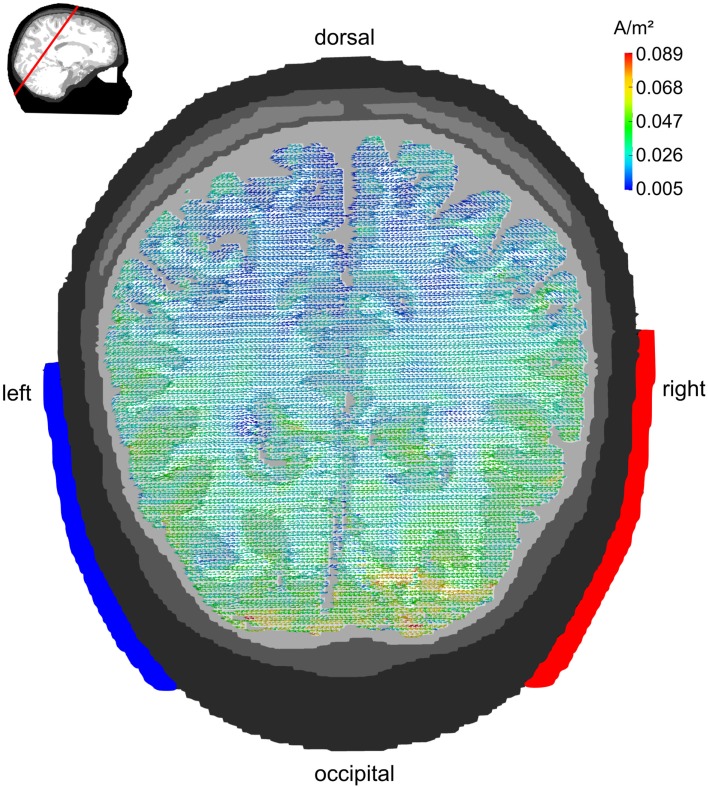
**Lateral configuration P7/P8: transversal view**. Stimulation electrodes are centered around electrode positions P7 and P8. The pattern of current densities shows a clear maximum over posterior brain regions. However, the current flow is not restricted to occipital cortex but reaches also parietal and temporal cortex. The embedded figure in the upper left corner depicts the section slice.

Figure 5 displays the current densities for electrodes being centered around 10–20 locations P7 and P8. Posterior brain areas receive strongest stimulation (up to 0.089 A/m^2^). However, also parietal and temporal cortex reach current densities in the range of 0.03–0.07 A/m^2^. Even some gyri of the frontal lobe that are close to CSF receive current densities up to 0.05 A/m^2^. The electrode montage is suited to stimulate occipital cortex. In contrast to the Cz/Oz montage, the current flow elicited by the P7/P8 montage is not limited to medial parts of the occipital cortex but reaches also the more lateral regions.

## Discussion

### Usability for occipital stimulation

As our simulation has demonstrated both, Cz/Oz and P7/P8 electrode montage are suited for stimulating the occipital cortex. This confirms the results of existing studies. Usually a Cz/Oz montage is used for tDCS (see Antal et al., 2008 for an overview) as well as for tACS (Kanai et al., 2008). But especially for tACS the problem of retinal phosphenes arises (Paulus, 2010). The closer one of the stimulation electrodes is to the eyes, the easier phosphenes are perceived. This especially holds true for the FPz/Oz configuration, an effect that our simulation confirms. An alternative would be the P7/P8 configuration which is far away from the eye balls and was successfully used with tACS by Zaehle et al. (2010). They adjusted the stimulation strength individually to assure that no phosphenes were elicited by the stimulation. Nevertheless, our simulation reveals that even with this montage, frontal areas might receive moderate electrical input. This makes it mandatory to stimulate the visual cortex below the individual phosphene threshold in order to avoid stimulation of the retina.

When applying tACS, modeling current flow reveals two different modes of stimulation. When two stimulation electrodes are placed at homologous location in the two hemispheres (e.g., P7/P8), this results in the two hemispheres being stimulated at 180° phase shift. Typically, brain oscillations are generated by two symmetrically located neural generators – one in each hemisphere (Chapman et al., 1984; Rodin and Rodin, 1995). Furthermore, two homologous electrodes in the two hemispheres usually oscillate without significant phase shift (Nikouline et al., 2001; Nunez et al., 2001). Since interhemispheric phase synchronization reflects functional coupling (Varela et al., 2001), cognitive functions that require the two oscillators to operate without phase shift could be disturbed by such an out of phase stimulation. Another type of stimulation would be achieved with electrodes being arranged along the midline (e.g., Cz/Oz). Here, the two lateral generators of an oscillation would be stimulated without phase shift.

### Usability for frontal stimulation

Our simulation clearly demonstrated that a midline configuration (FPz/Oz) is not suited to stimulate the frontal lobe. Due to the CSF in the interhemispheric cleft, current flow is rather non-focal. Therefore, a bilateral configuration (F7/F8) is advantageous. Firstly, a strong current flow in the frontal lobe is apparent and, secondly, the current flow is rather focal and other cortical areas receive no or weak current densities. Thirdly, current strength on both hemispheres is similar which makes a bilateral configuration especially suitable for the application in depression therapy. While some studies used a configuration with the anode over the left frontal lobe and the cathode over the right orbit (e.g., Fregni et al., 2006a; Boggio et al., 2008), a symmetrical configuration might be better suited. This way, the excitability of the left dlPFC could be enhanced and, simultaneously, the excitability of the right dlPFC could be reduced. Thus, tDCS could be more effective and at the same time lower stimulation intensities might be required to obtain the deserved effects. Another obvious aspect of our simulation is that within the frontal lobe the activation is rather widespread. Even if the dlPFC is the target of the stimulation it is difficult to argue that the observed effects are elicited by a selective modulation of the activity of the dlPFC. The same argument holds true for the stimulation of Broca’s area. Marangolo et al. (2011) stimulated three aphasic patients with brain lesions to different cortical structures functionally connected to Broca’s area. Although the same electrode configuration was used, all subjects exhibited improvement in speech production.

### General discussion

The analysis of the current densities in our realistically shaped finite-element model revealed the strongest current flow to be in the skin of the scalp. This effect is due to the better conductivity of skin as compared to bone and has been described previously for spherical (Miranda et al., 2006) and realistic head models (Salvador et al., 2010). In fact, the strong current density of more than 1 A/m^2^ is about 10 times stronger than that in brain tissue. Therefore, we had to disable the visualization of current flow in the scalp in order to see more subtle patterns of current densities inside the skull. Along the same lines, within the skull current densities in CSF were much stronger than in brain tissue. This effect is also due to the better conductivity of CSF as compared to gray or white matter and has been described before for realistic head models (Salvador et al., 2010). A further phenomenon was the pattern of current densities within gray and white matter. Generally speaking, current density was always stronger in tissue adjacent to CSF. Especially, gray matter structures (e.g., gyri) peaking into CSF resulted in strong current densities. CSF “shunts” the current flow so that nearby structures exhibit stronger current flow. This indicates that individual anatomical differences can have an effect of the current flow during TES. Thus, ideally one would want to compute individual head models to simulate the current flow for the individual patient to be stimulated (Halko et al., 2011). With this procedure, one could adjust the electrode positions and avoid inadequate stimulation sites. However, this requires MRI images of each patient and is computationally expensive and time consuming. Nevertheless, software solutions like the free SimNIBS (Windhoff et al., 2011) are already available.

**Figure 6:**
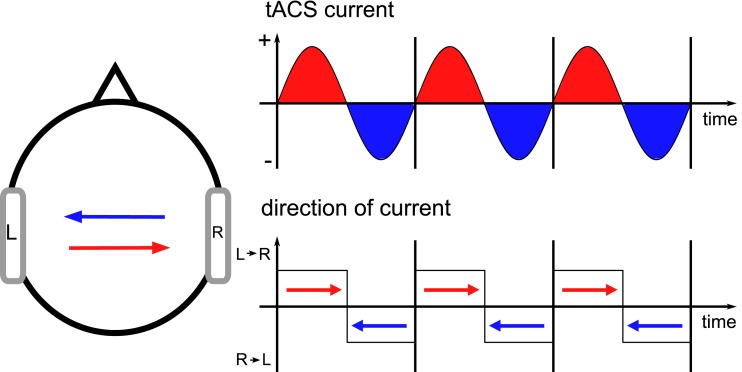
**Current flow during tACS**. When current is applied sinusoidally, the direction of current flow flips back and forth by 180° for every half-wave. Let us consider the left stimulation electrode. During the positive half-wave, it represents the anode of a tDCS stimulation with variable amplitude. During the negative half-wave, the direction of current flow suddenly flips by 180°. During each half-wave the direction remains stable but the strength of the current flow varies. Stimulation electrodes are depicted in gray.

In addition to tDCS, also tACS has recently been applied in therapy of neurological patients (Sabel et al., 2011). The idea of tACS is to interfere with brain oscillations which are known to be relevant for human cognition (Herrmann et al., 2004) and to be disturbed in some psychiatric and neurologic diseases (Herrmann and Demiralp, 2005; Uhlhaas et al., 2008). TACS is capable of enhancing the amplitude of ongoing brain oscillations (Zaehle et al., 2010). In our model, current flow was always from the anode to the cathode as can be seen from the direction of the cones that represent the direction of current flow. If anode and cathode are interchanged, this yields the same pattern of current densities. However, the direction of each cone of current flow flips by 180° (Wagner et al., 2007). For tACS, the direction of current flow flips back and forth for every half-wave of the stimulation (Figure 6).

Additionally, one has to take into account other possible electrode montages. Different electrode sizes (Nitsche et al., 2007), shapes (Datta et al., 2008, 2009), and number of electrodes (Feira et al., 2009) can help to overcome the limitation of the focality of TES. The simulation with modeling studies will help to make predictions about the outcome of a specific electrode montage with specific stimulation-parameters on a specific individual. Therefore, experiments have to follow to take the predictions into practice and eventually into therapy.

Our results raise the question whether weak currents applied with TES are able to influence the activity of cortical neurons in the human brain. In our modeling study we demonstrated that TES can lead to significant current flow inside the human cortex, despite a large amount of the current being short-circuited by the well-conducting skin (Holdefer et al., 2006). Intracranial electric stimulation of neurons in animals has demonstrated that axons and especially the axon hillock are sensitive to this kind of stimulation due to the high number of voltage-sensitive Na ion channels (Nowak and Bullier, 1998). Francis et al. (2003) were able to demonstrate that electric fields of 140 μV/mm are sufficient to increase the firing rate of single neurons (i.e., super-threshold stimulation). Miranda et al. (2006) used an isotropic spherical head model to demonstrate that 2.0 mA of tDCS results in 0.1 A/m^2^ corresponding to an electric field of 220 μV/mm. Our anisotropic simulation revealed current densities in the GM up to 0.1 A/m^2^. Dividing that value by the GM conductivity of 0.24 S/m reveals electric fields up to 417 μV/mm, which can be considered super-threshold. Additionally, one has to keep in mind that the current densities in the GM depend linearly on the total current. When the total current is doubled, the current densities in the GM will be 0.2 A/m^2^. Miranda et al. (2006) used a total current of 2.0 mA which is twice of what we used in our study. However, they used a significantly higher skin to skull conductivity ratio (75:1, while we used 43:1). Thus, major currents were short-circuited by the skin and minor currents penetrated the low conductive skull. Therefore, the current densities in the GM in both studies are comparable.

Modeling studies have elaborated on the effects of the size and position of the “return” electrode (Datta et al., 2010). They demonstrated that both parameters have a strong influence on the specificity of the stimulation and the current flow under the “stimulating” electrode. Furthermore, electrode locations are critical with regard to the amount of current shunted through the scalp (Miranda et al., 2006). Modeling studies can provide valuable insights about the general effects of the positions of the electrodes.

One has to keep in mind that simplified rules (e.g., anode – enhanced excitability, cathode – reduced excitability) can be misleading, because the distribution of the current flow through the head is much more complex. Common parameters of TES intensity (current intensity, duration of the stimulation, and overall electrode size) cannot predict the current that reaches the cortex. As other modeling studies demonstrated, the simulation of the current flow can help to define the correct tDCS intensity (Sadleir et al., 2010). A limitation of the usability of modeling approaches is represented by the parameters of the individuals, because cortical excitability is modulated by, for example, medication (Ziemann, 2003), which is especially relevant in clinical populations. This has to be considered when a study is conducted as it may affect the results.

Brunoni et al. (2012) concluded that the situation of TES is like the situation of TMS several years ago. A lot of studies have been conducted to explore the use of TES in therapy, but sample sizes were small and Phase III studies are still missing. We believe that modeling the current flow can help tDCS and tACS to reach therapeutic success in the future.

## Conflict of Interest Statement

The authors declare that the research was conducted in the absence of any commercial or financial relationships that could be construed as a potential conflict of interest.

## Footnotes

^1^http://www.fmrib.ox.ac.uk/fsl

^2^http://fsl.fmrib.ox.ac.uk/fsl/fdt/fdt_utils.html#vecreg

^3^http://www.mrt.uni-jena.de

^4^http://www.sci.utah.edu/cibc/software/106-scirun.html

## References

[B1] Akhtari M. Bryant H. C. Marnelak A. N. Flynn E. R. Heller L. Shih J. J. Mandelkern M. Matlachov A. Ranken D. M. Best E. D. DiMauro M. A. Lee R. R. Sutherling W. W. (2002). Conductivities of three-layer live human skull. Brain Topogr. 14, 151–167 10.1023/A:1014590923185 12002346

[B2] Antal A. Boros K. Poreisz C. Chaieb L. Terney D. Paulus W. (2008). Comparatively weak after-effects of transcranial alternating current stimulation (tACS) on cortical excitability in humans. Brain Stimul. 1, 97–105 10.1016/j.brs.2007.10.001 20633376

[B3] Antal A. Kincses T. Z. Nitsche M. A. Paulus W. (2003). Manipulation of phosphene thresholds by transcranial direct current stimulation in man. Exp. Brain Res. 150, 375–378 12698316 10.1007/s00221-003-1459-8

[B4] Antal A. Kinsces T. Z. Nitsche M. A. Bartfai O. Paulus W. (2004). Excitability changes induced in the human primary visual cortex by transcranial direct current stimulation: direct electrophysiological evidence. Invest. Ophthalmol. Vis. Sci. 45, 702–707 10.1167/iovs.03-0688 14744917

[B5] Antal A. Paulus W. (2008). Transcranial direct current stimulation and visual perception. Perception 37, 367–374 10.1068/p5872 18491714

[B6] Baker J. M. Rorden C. Fridriksson J. (2010). Using transcranial direct-current stimulation to treat stroke patients with aphasia. Stroke 41, 1229–1236 10.1161/STROKEAHA.109.569764 20395612 PMC2876210

[B7] Baumann S. B. Wozny D. R. Kelly S. K. Meno F. M. (1997). The electrical conductivity of human cerebrospinal fluid at body temperature. IEEE Trans. Biomed. Eng. 44, 220–223 10.1109/10.554770 9216137

[B8] Benninger D. H. Lomarev M. Lopez G. Wassermann E. M. Li X. Considine E. Hallett M. (2010). Transcranial direct current stimulation for the treatment of Parkinson’s disease. J. Neurol. Neurosurg. Psychiatr. 81, 1105–1111 10.1136/jnnp.2009.202556 20870863 PMC4162743

[B9] Bindman L. J. Lippold O. C. J. Redfearn J. W. T. (1964). The action of brief polarizing currents on the cerebral cortex of the rat (1) during current flow and (2) in the production of long-lasting after-effects. J. Physiol. (Lond.) 172, 369–382 14199369 10.1113/jphysiol.1964.sp007425 PMC1368854

[B10] Boggio P. S. Amancio E. J. Correa C. F. Cecilio S. Valasek C. Bajwa Z. Freedman S. D. Pascual-Leone A. Edwards D. J. Fregni F. (2009). Transcranial DC stimulation coupled with TENS for the treatment of chronic pain: a preliminary study. Clin. J. Pain 25, 691–695 10.1097/AJP.0b013e3181af1414 19920718

[B11] Boggio P. S. Ferrucci R. Rigonatti S. P. Covre P. Nitsche M. Pascual-Leone A. Fregni F. (2006). Effects of transcranial direct current stimulation on working memory in patients with Parkinson’s disease. J. Neurol. Sci. 249, 31–38 10.1016/j.jns.2006.05.062 16843494

[B12] Boggio P. S. Rigonatti S. P. Ribeiro R. B. Myczkowski M. L. Nitsche M. A. Pascual-Leone A. Fregni F. (2008). A randomized, doubleblind clinical trial on the efficacy of cortical direct current stimulation for the treatment of major depression. Int. J. Neuropsychopharmacol. 11, 249–254 10.1017/S1461145707007833 17559710 PMC3372849

[B13] Boggio P. S. Valasek C. A. Campanh ã C. Giglio A. C. Baptista N. I. Lapenta O. M. Fregni F. (2011). Non-invasive brain stimulation to assess and modulate neuroplasticity in Alzheimer’s disease. Neuropsychol. Rehabil. 21, 703–716 10.1080/09602011.2011.617943 21942868

[B14] Brunoni A. R. Nitsche M. A. Bolognini N. Bikson M. Wagner T. Merabet L. Edwards D. J. Valero-Cabre A. Rotenberg A. Pascual-Leone A. Ferrucci R. Priori A. Boggio P. S. Fregni F. (2012). Clinical research with transcranial direct current stimulation (tDCS): challenges and future directions. Brain Stimul. 5, 175–195 10.1016/j.brs.2011.03.002 22037126 PMC3270156

[B15] Chaieb L. Antal A. Paulus W. (2008). Gender-specific modulation of short-term neuroplasticity in the visual cortex induced by transcranial direct current stimulation. Vis. Neurosci. 25, 77–81 10.1017/S0952523808080097 18282312

[B16] Chapman R. M. Ilmoniemi R. J. Barbanera S. Romani G. L. (1984). Selective localization of alpha brain activity with neuromagnetic measurements. Electroencephalogr. Clin. Neurophysiol. 58, 569–572 10.1016/0013-4694(84)90047-6 6209107

[B17] Dannhauer M. Lanfer B. Wolters C. H. Knösche T. R. (2011). Modeling of the human skull in EEG source analysis. Hum. Brain Mapp. 32, 1383–1399 10.1002/hbm.21114 20690140 PMC6869856

[B18] Datta A. Bansal V. Diaz J. Patel J. Reato D. Bikson M. (2009). Gyri-precise head model of transcranial direct current stimulation: Improved spatial focality using a ring electrode versus conventional rectangular pad. Brain Stimul. 2, 201–207 10.1016/j.brs.2009.03.005 PMC2790295 20648973

[B19] Datta A. Elwassif M. Battaglia F. Bikson M. (2008). Transcranial current stimulation focality using disc and ring electrode configurations. J. Neural Eng. 5, 163–174 10.1088/1741-2560/5/2/007 18441418

[B20] Datta A. Scaturro A. R. J. Bikson M. (2010). Electrode montages for tDCS and weak transcranial electrical stimulation role of ’return’ electrode’s position and size. Clin. Neurophysiol. 121, 1976–1978 10.1016/j.clinph.2010.05.020 21035740 PMC2983105

[B21] Dell’Osso B. Zanoni S. Ferrucci R. Vergari M. Castellano F. D’Urso N. Dobrea C. Benatti B. Arici C. Priori A. Altamura A. C. (2011). Transcranial direct current stimulation for the outpatient treatment of poor-responder depressed patients. Eur. Psychiatry. 10.1016/j.eurpsy.2011.02.008 21621982

[B22] Edwards D. J. Krebs H. I. Rykman A. Zipse J. Thickbroom G. W. Mastaglia F. L. Pascual-Leone A. Volpe B. T. (2009). Raised corticomotor excitability of M1 forearm area following anodal tDCS is sustained during robotic wrist therapy in chronic stroke. Restor. Neurol. Neurosci. 27, 199–207 19531875 10.3233/RNN-2009-0470 PMC4510929

[B23] Feira P. Leal A. Miranda P. C. (2009). “Comparing different electrode configurations using the 10-10 international system in tDCS: a finite element method analysis,” in 31st Annual International Conference of the IEEE Engineering in Medicine and Biology Society, Minneapolis, 1596–1599 10.1109/IEMBS.2009.5334121 19964541

[B24] Ferrucci R. Mameli F. Guidi I. Mrakic-Sposta S. Vergari M. Marceglia S. Cogiamanian F. Barbieri S. Scarpini E. Priori A. (2008). Transcranial direct current stimulation improves recognition memory in Alzheimer disease. Neurology 71, 493–498 10.1212/01.wnl.0000317060.43722.a3 18525028

[B25] Francis J. Gluckman B. J. Schiff S. J. (2003). Sensitivity of neurons to weak electric fields. J. Neurosci. 23, 7255–7261 12917358 10.1523/JNEUROSCI.23-19-07255.2003 PMC6740448

[B26] Fregni F. Boggio P. S. Nitsche M. A. Rigonatti S. P. Pascual-Leone A. (2006a). Cognitive effects of repeated sessions of transcranial direct current stimulation in patients with depression. Depress. Anxiety 23, 482–484 10.1002/da.20201 16845648

[B27] Fregni F. Thome-Souza S. Nitsche M. A. Freedman S. D. Valente K. D. Pascual-Leone A. (2006b). A controlled clinical trial of cathodal DC polarization in patients with refractory epilepsy. Epilepsia 47, 335–342 10.1111/j.1528-1167.2006.00426.x 16499758

[B28] Fridriksson J. Richardson J. D. Baker J. M. Rorden C. (2011). Transcranial direct current stimulation improves naming reaction time in fluent aphasia: a double-blind, sham-controlled study. Stroke 42, 819–821 10.1161/STROKEAHA.110.600288 21233468 PMC8210639

[B29] George M. S. Aston-Jones G. (2010). Noninvasive techniques for probing neurocircuitry and treating illness: vagus nerve stimulation (VNS), transcranial magnetic stimulation (TMS) and transcranial direct current stimulation (tDCS). Neuropsychopharmacology 35, 301–316 10.1038/npp.2009.87 19693003 PMC3055429

[B30] Grimm S. Beck J. Schuepbach D. Hell D. Boesiger P. Bermpohl F. Niehaus L. Boeker H. Northoff G. (2008). Imbalance between left and right dorsolateral prefrontal cortex in major depression is linked to negative emotional judgment: an fMRI study in severe major depressive disorder. Biol. Psychiatry 63, 369–376 10.1016/j.biopsych.2007.05.033 17888408

[B31] Halko M. A. Datta A. Plow E. B. Scaturro J. Bikson M. Merabet L. B. (2011). Neuroplastic changes following rehabilitative training correlate with regional electric field induced with tDCS. Neuroimage 57, 885–891 10.1016/j.neuroimage.2011.05.026 21620985 PMC3167218

[B32] Heimrath K. Sandmann P. Becke A. Müller N. G. Zaehle T. (2012). Behavioral and electrophysiological effects of transcranial direct current stimulation (tDCS) of the parietal cortex in a visuo-spatial working memory task. Front Psychiatry 3:56 10.3389/fpsyt.2012.00056 22723784 PMC3378949

[B33] Herrmann C. S. Demiralp T. (2005). Human EEG gamma oscillations in neuropsychiatric disorders. Clin. Neurophysiol. 116, 2719–2733 10.1016/j.clinph.2005.07.007 16253555

[B34] Herrmann C. S. Grigutsch M. Busch N. (2004). “EEG oscillations and wavelet analysis,” in Event-Related Potentials: A Methods Handbook, ed. Handy T. C. (Cambridge, MA: Bradford Book), 229–259

[B35] Hillis A. E. Work M. Barker P. B. Jacobs P. B. Breese E. L. Maurer K. (2004). Reexamining the brain regions crucial for orchestrating speech articulation. Brain 127, 1479–1487 10.1093/brain/awh172 15090478

[B36] Holdefer R. Sadleir R. Russell M. (2006). Predicted current densities in the brain during transcranial electrical stimulation. Clin. Neurophysiol. 117, 1388–1397 10.1016/j.clinph.2006.02.020 16644273 PMC2426751

[B37] Holland R. Leff A. P. Josephs O. Galea J. M. Desikan M. Price C. J. Rothwell J. C. Crinion J. (2011). Speech facilitation by left inferior frontal cortex stimulation. Curr. Biol. 21, 1403–1407 10.1016/j.cub.2011.07.021 21820308 PMC3315006

[B38] Hummel F. Celnik P. Giraux P. Floel A. Wu W.-H. Gerloff C. Cohen L. G. (2005). Effects of non-invasive cortical stimulation on skilled motor function in chronic stroke. Brain 128, 490–499 10.1093/brain/awh369 15634731

[B39] Jenkinson M. Pechaud M. Smith S. (2005). “BET2: MR-based estimation of brain, skull and scalp surfaces,” in Eleventh Annual Meeting of the Organization for Human Brain Mapping, Toronto

[B40] Jenkinson M. Smith S. (2001). A global optimisation method for robust affine registration of brain images. Med. Image Anal. 5, 143–156 10.1016/S1361-8415(01)00036-6 11516708

[B41] Kalu U. G. Sexton C. E. Loo C. K. Ebmeier K. P. (2012). Transcranial direct current stimulation in the treatment of major depression: a meta-analysis. Psychol. Med. 12, 1–10 10.1017/S0033291711003059 22236735

[B42] Kanai R. Chaieb L. Antal A. Walsh V. Paulus W. (2008). Frequency-dependent electrical stimulation of the visual cortex. Curr. Biol. 18, 1839–1843 10.1016/j.cub.2008.10.027 19026538

[B43] Lew S. Wolters C. H. Röer C. Dierkes T. MacLeod R. S. (2009). Accuracy and run-time comparison for different potential approaches and iterative solvers in finite element method based EEG source analysis. Appl. Numer. Math. 59, 1970–1988 10.1016/j.apnum.2009.02.006 20161462 PMC2791331

[B44] Marangolo P. Marinelli C. V. Bonifazi S. Fiori V. Ceravolo M. G. Tomaiuolo L. P. F. (2011). Electrical stimulation over the left inferior frontal gyrus (IFG) determines long-term effects in the recovery of speech apraxia in three chronic aphasics. Behav. Brain Res. 225, 498–504 10.1016/j.bbr.2011.08.008 21856336

[B45] Marshall L. Helgadottir H. Mölle M. Born J. (2006). Boosting slow oscillations during sleep potentiates memory. Nature 444, 610–613 10.1038/nature05278 17086200

[B46] Miranda P. C. Lomarev M. Hallett M. (2006). Modeling the current distribution during transcranial direct current stimulation. Clin. Neurophysiol. 117, 1623–1629 10.1016/j.clinph.2006.04.009 16762592

[B47] Monti A. Cogiamanian F. Marceglia S. Ferrucci R. Mameli F. Mrakic-Sposta S. Vergari M. Zago S. Priori A. (2008). Improved naming after transcranial direct current stimulation in aphasia. J. Neurol. Neurosurg. Psychiatr. 79, 451–453 10.1136/jnnp.2007.135277 18096677

[B48] Nikouline V. V. Linkenkaer-Hansen K. Huttunen J. Ilmoniemi R. J. (2001). Interhemispheric phase synchrony and amplitude correlation of spontaneous beta oscillations in human subjects: a magnetoencephalographic study. Neuroreport 12, 2487–2491 10.1097/00001756-200108080-00040 11496135

[B49] Nitsche M. A. Doemkes S. Karaköse T. Antal A. Liebetanz D. Lang N. Tergau F. Paulus W. (2007). Shaping the effects of transcranial direct current stimulation of the human motor cortex. J. Neurophysiol. 97, 3109–3117 10.1152/jn.01312.2006 17251360

[B50] Nitsche M. A. Paulus W. (2000). Excitability changes induced in the human motor cortex by weak transcranial direct current stimulation. J. Physiol. 527, 633–639 10.1111/j.1469-7793.2000.t01-1-00633.x 10990547 PMC2270099

[B51] Nitsche M. A. Paulus W. (2001). Sustained excitability elevations induced by transcranial dc motor cortex stimulation in humans. Neurology 57, 1899–1901 10.1212/WNL.57.10.1899 11723286

[B52] Nitsche M. A. Paulus W. (2009). Noninvasive brain stimulation protocols in the treatment of epilepsy: current state and perspectives. Neurotherapeutics 6, 244–250 10.1016/j.nurt.2009.01.003 19332316 PMC5084200

[B53] Nowak L. G. Bullier J. (1998). Axons, but not cell bodies, are activated by electrical stimulation in cortical gray matter I. Evidence from chronaxie measurements. Exp. Brain Res. 118, 477–488 10.1007/s002210050304 9504843

[B54] Nunez P. L. Wingeier B. M. Silberstein R. B. (2001). Spatial-temporal structures of human alpha rhythms: theory, microcurrent sources, multiscale measurements, and global binding of local networks. Hum. Brain Mapp. 13, 125–164 10.1002/hbm.1030 11376500 PMC6872048

[B55] Olesch J. Ruthotto L. Kugel H. Skare S. Fischer B. Wolters C. H. (2010). A variational approach for the correction of field-inhomogeneities in EPI sequences. SPIE Med. Imag. Image Process. 7623, 1–8

[B56] Paulus W. (2010). On the difficulties of separating retinal from cortical origins of phosphenes when using transcranial alternating current stimulation (tACS). Clin Neurophysiol 121, 987–991 10.1016/S1388-2457(10)60132-0 20181514

[B57] Paulus W. (2011). Transcranial electrical stimulation (tES – tDCS; tRNS, tACS) methods. Neuropsychol. Rehab. 21, 602–617 10.1080/09602011.2011.557292 21819181

[B58] Plonsey R. Heppner D. (1967). Considerations on quasi-stationarity in electro-physiological systems. Bull. Math. Biophys. 29, 657–664 10.1007/BF02476917 5582145

[B59] Rigonatti S. P. Boggio P. S. Myczkowski M. L. Otta E. Fiquer J. T. Ribeiro R. B. Nitsche M. A. Pascual-Leone A. Fregni F. (2008). Transcranial direct stimulation and fluoxetine for the treatment of depression. Eur. Psychiatry 23, 74–76 10.1016/j.eurpsy.2008.01.267 18023968

[B60] Rodin E. A. Rodin M. J. (1995). Dipole sources of the human alpha rhythm. Brain Topogr. 7, 201–208 10.1007/BF01202379 7599019

[B61] Rullmann M. Anwander A. Dannhauer M. Warfield S. Duffy F. Wolters C. (2009). EEG source analysis of epileptiform activity using a 1 mm anisotropic hexahedra finite element head model. Neuroimage 44, 399–410 10.1016/j.neuroimage.2008.09.009 18848896 PMC2642992

[B62] Sabel B. A. Fedorov A. B. Naue N. Borrmanna A. Herrmann C. Gall C. (2011). Non-invasive alternating current stimulation improves vision in optic neuropathy. Restor. Neurol. Neurosci. 29, 493–505 22124039 10.3233/RNN-2011-0624

[B63] Sadleir R. J. Vannorsdall T. D. Schretlen D. J. Gordon B. (2010). Transcranial direct current stimulation (tDCS) in a realistic head model. Neuroimage 51, 1310–1318 10.1016/j.neuroimage.2010.03.052 20350607

[B64] Salvador R. Mekonnen A. Ruffini G. Miranda P. C. (2010). Modeling the electric field induced in a high resolution realistic head model during transcranial current stimulation. Conf. Proc. IEEE Eng. Med. Biol. Soc. 2010, 2073–2076 21095946 10.1109/IEMBS.2010.5626315

[B65] Smith S. M. (2002). Fast robust automated brain extraction. Hum. Brain Mapp. 17, 143–155 10.1002/hbm.10062 12391568 PMC6871816

[B66] Tuch D. S. Wedeen V. J. Dale A. M. George J. S. Belliveau J. W. (2001). Conductivity tensor mapping of the human brain using diffusion tensor MRI. Proc. Natl. Acad. Sci. U.S.A. 98, 11697–11701 10.1073/pnas.171473898 11573005 PMC58792

[B67] Uhlhaas P. J. Haenschel C. Nikolic D. Singer W. (2008). The role of oscillations and synchrony in cortical networks and their putative relevance for the pathophysiology of schizophrenia. Schizophr. Bull. 34, 927–943 10.1093/schbul/sbn062 18562344 PMC2632472

[B68] Varela F. Lachaux J. P. Rodriguez E. Martinerie J. (2001). The brainweb: phase synchronization and large-scale integration. Nat. Rev. Neurosci. 2, 229–239 10.1038/35067042 11283746

[B69] Wagner T. Fregni F. Fectau S. Grodzinsky A. Zahn M. Pascual-Leone A. (2007). Transcranial direct current stimulation: a computer-based human model study. Neuroimage 35, 1113–1124 10.1016/j.neuroimage.2007.01.027 17337213

[B70] Windhoff M. Opitz A. Thielscher A. (2011). Electric field calculations in brain stimulation based on finite elements: an optimized processing pipeline for the generation and usage of accurate individual head models. Hum. Brain Mapp. 10.1002/hbm.21479 22109746 PMC6870291

[B71] Wolters C. H. Anwander A. Berti G. Hartmann U. (2007a). Geometry-adapted hexahedral meshes improve accuracy of finite element method based EEG source analysis. IEEE Trans. Biomed. Eng. 54, 1446–1453 10.1109/TBME.2007.890736 17694865

[B72] Wolters C. H. Köstler H. Möller C. Härdtlein J. Grasedyck L. Hackbusch W. (2007b). Numerical mathematics of the subtraction method for the modeling of a current dipole in EEG source reconstruction using finite element head models. SIAM J. Sci. Comput. 30, 24–45 10.1137/060659053

[B73] Wolters C. H. Kuhn M. Anwander A. Reitzinger S. (2002). A parallel algebraic multigrid solver for finite element method based source localization in the human brain. Comput. Vis. Sci. 5, 165–177 10.1007/s00791-002-0098-0

[B74] Zaehle T. Rach S. Herrmann C. S. (2010). Transcranial alternating current stimulation enhances individual alpha activity in human EEG. PLoS ONE 5, e13766 10.1371/journal.pone.0013766 21072168 PMC2967471

[B75] Zaehle T. Sandmann P. Thorne J. D. Jäncke L. Herrmann C. S. (2011). Transcranial direct current stimulation of the prefrontal cortex modulates working memory performance: combined behavioural and electrophysiological evidence. BMC Neurosci. 12, 1–11 10.1186/1471-2202-12-2 21211016 PMC3024225

[B76] Zhang Y. Brady M. Smith S. (2001). Segmentation of brain MR images through a hidden markov random field model and the expectation maximization algorithm. IEEE Trans. Med. Imaging 20, 45–57 10.1109/42.906424 11293691

[B77] Ziemann U. (2003). Pharmacology of TMS. Suppl. Clin. Neurophysiol. 56, 226–231 10.1016/S1567-424X(09)70226-0 14677399

